# Idiopathic Extremity Arteriovenous Fistula: A Rare Etiology of Cardiac Failure

**DOI:** 10.7759/cureus.28459

**Published:** 2022-08-27

**Authors:** Anushka C Ninama, Shreyas Bellur, Sreekar Balasundaram

**Affiliations:** 1 Department of Surgery, St. John's Medical College Hospital, Bangalore, IND; 2 Department of Cardiothoracic Surgery, St. John’s Medical College Hospital, Bangalore, IND

**Keywords:** vascular, heart failure, idiopathic, arterio-venous fistula, extremity

## Abstract

Idiopathic extremity arteriovenous fistulas are rare, abnormal connections between arteries and veins commonly occurring between iliac vessels. We present the case of a 42-year-old female who was referred to our center for a mitral valve replacement with a one-year history of breathlessness and a six-month history of progressive abdominal distension. Physical examination revealed a pansystolic murmur in the mitral area, basal crepitations, and a non-tender pulsatile swelling in the right iliac fossa. Imaging showed severe mitral regurgitation in heart failure along with a large arteriovenous fistula between the common femoral vessels which suggested that the arteriovenous fistula was the etiology of heart failure. Postoperatively, the patient showed significant improvement in symptoms with imaging. On follow-up, the patient reported no progression of symptoms. Our case demonstrated an idiopathic extremity arteriovenous fistula in an unusual location, unexpectedly causing heart failure despite a significant valvular disease. Therefore, arteriovenous fistulas warrant inclusion in the differential of heart failure despite a valvular lesion and absence of classical signs of high output heart failure, as in our case.

## Introduction

Arteriovenous fistulas (AVFs) are high flow connections between arteries and veins with a variable etiology, most of them being surgically created extremity AVFs in those with chronic kidney disease (CKD) [1,2]. Firearm and stab injuries are the most common causes of AVFs [1,3].

Spontaneous unilateral idiopathic extremity AVFs are rare and mostly occur in the iliac vessels [2,3]. They have been found to be associated with the rupture of atherosclerotic aneurysms [1]. Even though there are not many cases reported in the literature, AVFs cannot be overlooked as they can lead to sequelae like congestive heart failure and loss of limb due to reduced circulation. A study by Kelm et al. found the incidence of iatrogenic AVFs to be 0.86%, with risk factors including high heparin dosage, commanding therapy, puncture of the left groin, arterial hypertension, and female gender [4]. Patients with failure-caused AVFs are found to have left ventricular dilatation and hypertrophy [5]. A study conducted by De Lima et al. on chronic kidney disease patients with AVFs showed echocardiographic changes corresponding to these changes [6]. Specific clinical findings to be looked out for are pulsatile mass along with a thrill and bruit, ischemia of the extremity, and asymmetric venous engorgement [2]. Peripheral pulses may or may not be palpable depending on the extent of the fistula. 

We report a 42-year-old female with idiopathic extremity AVFs presenting as a high output cardiac failure.

## Case presentation

A 42-year-old female with no known comorbidities was referred to our outpatient department for a mitral valve replacement with a one-year history of breathlessness associated with orthopnea, and for six months, progressive abdominal distension and subsequent pedal edema three months later. She had no history of trauma or catheter intervention. Her medical history was unremarkable apart from an uncomplicated lower segment Caesarian section 12 years ago.

Physical examination was significant for a holosystolic murmur heard in the mitral area, apex beat displacement, and basal crepitations. Further, inspection revealed a soft, immobile, non-tender pulsatile mass in the right groin region measuring around 10x5 cm in size associated with a thrill. On occlusion of the swelling, there was a rapid decrease in heart rate and a rise in blood pressure. We made a clinical diagnosis of severe mitral regurgitation in heart failure. 

**Figure 1:**
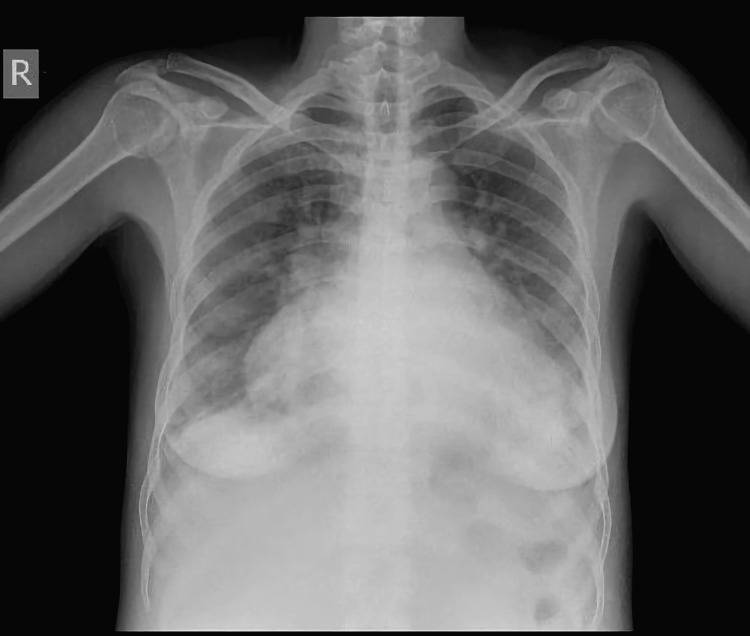
Preoperative chest X-ray

Laboratory investigations were unremarkable except for an increased thyroid stimulating hormone (TSH). The chest X-ray showed a cardiothoracic ratio of 0.78 (Figure 1), and the echocardiogram (ECHO) showed severe mitral regurgitation with all cardiac chambers dilated with preserved ventricular function and ejection fraction of 59%. 

**Figure 2:**
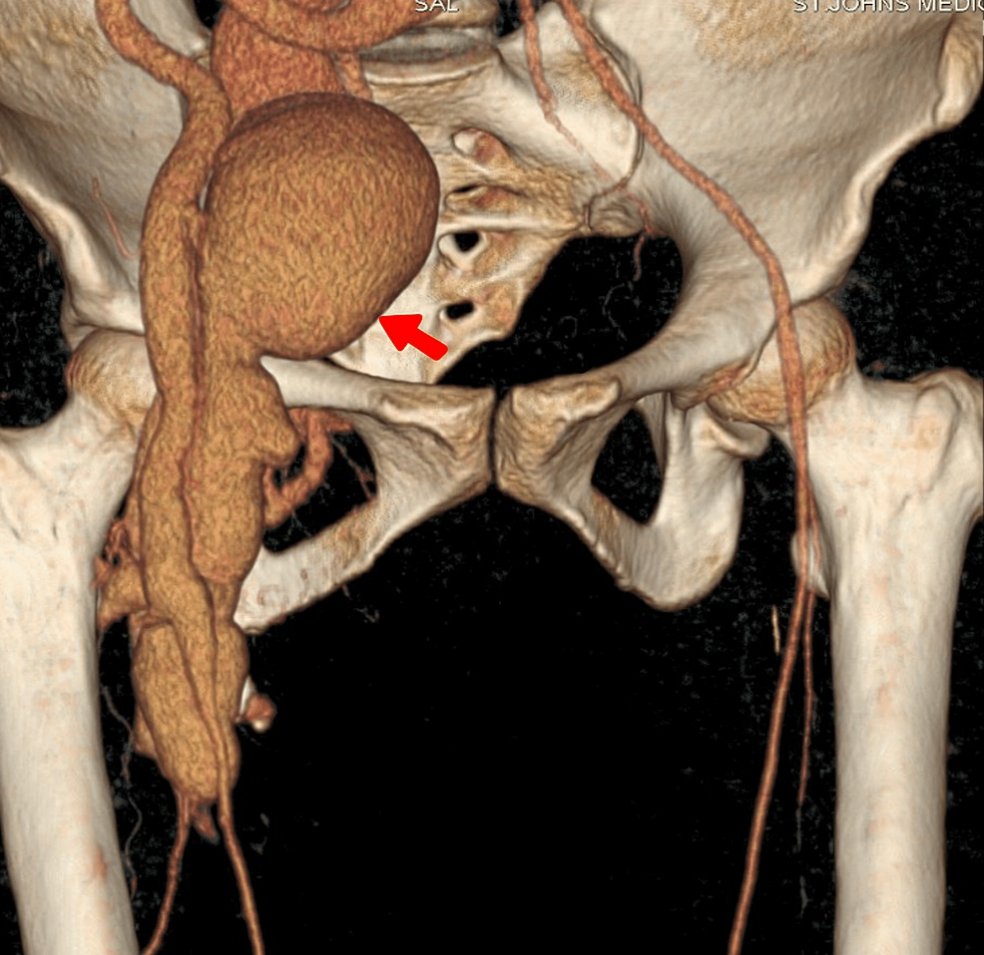
Arteriovenous fistula (indicated with an arrow) connection on CT of the abdomen and pelvis

Preoperatively, an ultrasound of the abdomen and pelvis suggested a possible right iliac artery aneurysm with a dilated inferior vena cava and moderate ascites, necessitating further imaging. Further, computed tomography (CT) of the chest and abdomen revealed a wide neck fistulous communication between the right common femoral vein (CFV) and the common femoral artery (CFA) with focal venous ectasia measuring 7x7 cm with no evidence of arterial occlusion or aneurysm (Figure 2). This suggested that the AVF was the cause of the heart failure, given preserved ventricular function. On CT, the right iliac artery was found to be normal, ruling out an aneurysm.

We counseled her for a surgical repair, and intraoperatively, we found an AVF between the common femoral artery and common femoral vein with numerous collaterals from the profunda femoris with a fistula opening of 2.5 cm. Collaterals were ligated, and the CFA and CFV were separated and repaired separately with 5-0 polypropylene. 

**Figure 3:**
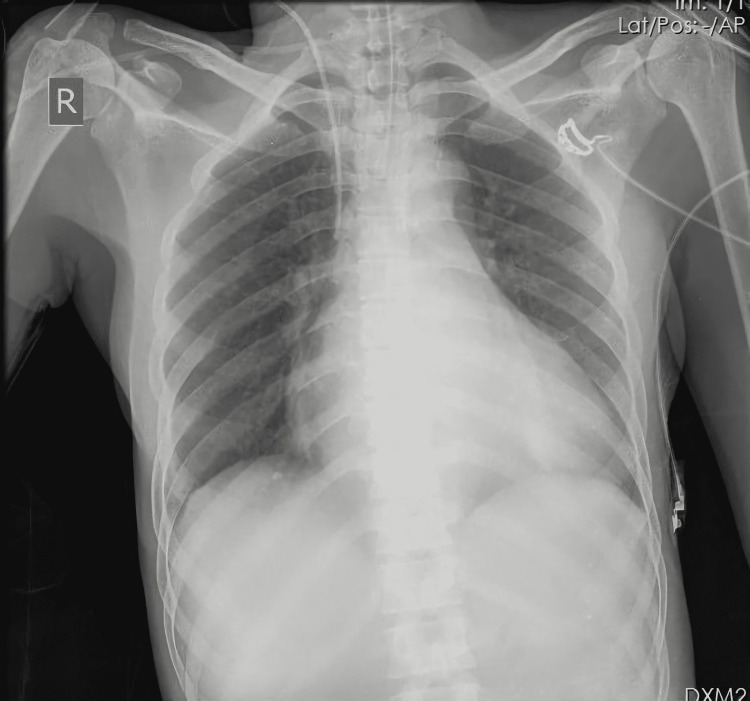
Postoperative chest X-ray

Postoperatively, the patient showed marked improvement in symptoms. Chest X-ray showed a considerable reduction in the cardiothoracic ratio falling from 0.78 to 0.69 (Figure 3). The echocardiogram revealed a reduction in the size of the cardiac chambers with moderate mitral regurgitation. Further hospital stay was uneventful, and she was discharged on postoperative day eight. On repeat follow-ups for six months, she reported no progression of symptoms.

## Discussion

Arteriovenous fistulas are abnormal connections between arteries and venous. Based on the etiology, they classify as congenital, traumatic, iatrogenic, and spontaneous AVFs [2]. The pathogenesis of an AVF is associated with shear stress, as evidenced by its association with atherosclerosis [7]. Most extremity AVFs are iatrogenically created for hemodialysis in patients with chronic kidney disease [1,2]. Patients with infections, malignancies, and congenital syndromes like Kippel-Trenaunay have also been demonstrated to have AV fistulas [8]. However, we could not establish the cause in our patient. There was no history since birth or evidence of chronic kidney disease requiring dialysis. Due to the lack of any other predisposing factors, we speculate that our patient's AVF was idiopathic in nature.

Clinically, peripheral AVFs present as pulsatile swellings with edema and hypertrophy of the affected limb [9]. There can also be a local rise in temperature. On occasion, peripheral pulses may be absent and are usually associated with a cold limb [3]. The persistence of an AVF eventually leads to dilation and weakening of the vessel wall, ultimately leading to high output heart failure [9,10]. Our patient presented with findings consistent with the literature. However, we initially attributed her heart failure to severe mitral regurgitation as there were no classical signs of high output heart failure on examination - wide pulse pressure and reduced systemic vascular resistance. Further, heart failure commonly occurs with upper limb AVFs and those closer to the heart [11].

Diagnosis of an AVF is clinical and supported by imaging such as duplex ultrasonography and CT angiogram [10]. We suggest a Doppler ultrasound initially and a CT angiogram if inconclusive. 

Surgical repair is the mainstay for all symptomatic AVFs consisting of resecting the fistula followed by primary closure or patch repair [8]. Expected complications of the procedure include significant hemorrhage and a groin infection [12]. After undergoing repair, our patient's heart failure symptoms markedly improved. This substantiated that the AVF was causing heart failure and was supported by the ECHO and chest x-ray findings. Interestingly, the AVF was responsible for heart failure despite not causing any local symptoms. Studies reported by MacRae et al. and Ozyüksel et al. showed similar findings in their case, wherein there was reduced left ventricular dilatation after removal of the AVF [5,10].

## Conclusions

Idiopathic extremity AVFs are rare entities that seldom cause heart failure. However, they warrant inclusion in the differential when an extremity swelling is present. Diagnosis is made by Doppler ultrasound and can be aided by a contrast-enhanced CT. Surgical resection of the fistula tract with primary closure is the treatment of choice.
